# Properdin Pattern Recognition on Proximal Tubular Cells Is Heparan Sulfate/Syndecan-1 but Not C3b Dependent and Can Be Blocked by Tick Protein Salp20

**DOI:** 10.3389/fimmu.2020.01643

**Published:** 2020-08-07

**Authors:** Rosa G. M. Lammerts, Ditmer T. Talsma, Wendy A. Dam, Mohamed R. Daha, Marc A. J. Seelen, Stefan P. Berger, Jacob van den Born

**Affiliations:** Department of Internal Medicine, Division of Nephrology, University Medical Center Groningen, University of Groningen, Groningen, Netherlands

**Keywords:** complement, properdin, C3, Salp20, syndecan-1

## Abstract

**Introduction:** Proteinuria contributes to progression of renal damage, partly by complement activation on proximal tubular epithelial cells. By pattern recognition, properdin has shown to bind to heparan sulfate proteoglycans on tubular epithelium and can initiate the alternative complement pathway (AP). Properdin however, also binds to C3b(Bb) and properdin binding to tubular cells might be influenced by the presence of C3b(Bb) on tubular cells and/or by variability in properdin proteins *in vitro*. In this study we carefully evaluated the specificity of the properdin – heparan sulfate interaction and whether this interaction could be exploited in order to block alternative complement activation.

**Methods:** Binding of various properdin preparations to proximal tubular epithelial cells (PTEC) and subsequent AP activation was determined in the presence or absence of C3 inhibitor Compstatin and properdin inhibitor Salp20. Heparan sulfate proteoglycan dependency of the pattern recognition of properdin was evaluated on PTEC knocked down for syndecan-1 by shRNA technology. Solid phase binding assays were used to evaluate the effectivity of heparin(oids) and recombinant Salp20 to block the pattern recognition of properdin.

**Results:** Binding of serum-derived and recombinant properdin preparations to PTECs could be dose-dependently inhibited (*P* < 0.01) and competed off (*P* < 0.01) by recombinant Salp20 (IC50: ~125 ng/ml) but not by Compstatin. Subsequent properdin-mediated AP activation on PTECs could be inhibited by Compstatin (*P* < 0.01) and blocked by recombinant Salp20 (*P* < 0.05). Syndecan-1 deficiency in PTECs resulted in a ~75% reduction of properdin binding (*P* = 0.057). In solid-phase binding assays, properdin binding to C3b could be dose-dependently inhibited by recombinant Salp20> heparin(oid) > C3b.

**Discussion:** In this study we showed that all properdin preparations recognize heparan sulfate/syndecan-1 on PTECs with and without Compstatin C3 blocking conditions. In contrast to Compstatin, recombinant Salp20 prevents heparan sulfate pattern recognition by properdin on PTECs. Both complement inhibitors prevented properdin-mediated C3 activation. Binding of properdin to C3b could also be blocked by heparin(oids) and recombinant Salp20. This work indicates that properdin serves as a docking station for AP activation on PTECs and a Salp20 analog or heparinoids may be viable inhibitors in properdin mediated AP activation.

## Introduction

Proteinuria is caused by the passage of proteins through the damaged glomerular filtration barrier and is an independent prognostic factor for the progression of chronic renal failure to end stage renal disease (1). Several mechanisms have been postulated on how proteinuria causes renal damage, one of them being via tubular complement activation. Evidence for involvement of the complement system in renal damage was already shown in 1985 by the finding of C3 deposits on the proximal tubular epithelial cells (PTECs) of nephrotic patients (2). Ultrafiltration of complement factors under proteinuric conditions may lead to alternative pathway activation within the renal tubules. This may be explained by the absence of a number of complement regulatory proteins on the apical membrane including decay accelerating factor (DAF), complement receptor 1 (CR1), and membrane cofactor protein (MCP) (3, 4) Complement regulatory protein CD59 is present on the brush border of proximal tubules, albeit weakly expressed (4). As a result, failure to downregulate the complement cascade might lead to tubular epithelial damage under proteinuric conditions.

The complement system consists of three pathways; the lectin pathway (LP), classical pathway (CP) and alternative pathway (AP). The LP and CP are initiated by pattern recognition molecules (e.g., MBL and C1q), whereas the current conception of the AP is thought to be a purely auto-activating route, via the spontaneous or induced formation of fluid-phase AP C3 convertase (5, 6). The three pathways merge at the formation of a C3 convertase, the major enzymes of the cascade (7, 8). For the CP and LP this is the C4bC2a complex, whilst in the AP the C3bBb complex is formed. The C3bBb complex is relatively unstable in plasma and requires stabilization by properdin, the only known positive regulator of the complement system (9). Properdin consists of seven thrombospondin repeat domains TSR0-TSR6 beginning at the N-terminus (10). However, understanding the complex biology of properdin has proven to be difficult due to the different sources of properdin used in biochemical studies and also its intricate self-associations. Through head-to-tail interactions of monomeric subunits, properdin can form cyclic dimers, trimers and tetramers under physiological conditions (11, 12). Additionally, non-physiological high molecular weight polymers can also form during long term storage and freeze/thaw cycles (12, 13). Moreover, stored properdin in the granules of neutrophils, which is released upon cell stimulation, may be structurally different than serum properdin either in its multimeric structure or in its posttranslational modifications (14, 15).

In the AP auto-activating theory it was thought that stabilizing the C3bBb complex was the only function of properdin. However, in the past decade data has accumulated stating that properdin can act as a pattern recognition molecule on PTECs, apoptotic, necrotic and bacterial cells (9). As ligands for properdin DNA and glycosaminoglycans have been proposed (16, 17). However, this theory was questioned by Harboe et al. since they showed that properdin binding to granulocyte MPO, endothelial cells and *Neisseria Meningitidis* is completely dependent on initial C3b binding, raising doubt on the conclusions of formerly published work (18). Their conclusion was based on properdin binding experiments in the presence or absence of Compstatin (18), a circular peptide inhibiting the cleavage of C3 into C3a and C3b. On the other hand, studies by the group of Van Kooten et al. in mice demonstrated that properdin can be found in glomeruli of C3 knockout mice during anti glomerular basement membrane disease indicating that C3 is not essential for properdin binding to tissues (19).

In proteinuric patients, AP activation has been linked to the presence of properdin on the PTEC brush border and *in vitro* binding of properdin and subsequent complement activation on HK-2 cells has also been shown. However, this is not the case for endothelial cells (20). In addition, urinary properdin excretion is associated with renal complement activation and worsening renal function (21, 22). More recently, our group showed that the binding of properdin to HK-2 cells is dependent on heparan sulfates (HS), since pretreatment of the cells with heparitinase abolished the binding of properdin (23). Moreover, competition experiments with heparin and non-anticoagulant heparinoids could reduce the binding of properdin to HK-2 cells, showing the treatment potential of heparinoids in AP mediated proteinuric damage (24). Co-localization of properdin with syndecan-1 on PTECs in an adriamycin induced nephropathy model suggested a role for the heparan sulfated proteoglycan (HSPG) syndecan-1 in the tubular binding of properdin (23). Syndecan-1 is a major membrane spanning HSPG in epithelial cells and has been shown to be upregulated on tubular epithelium in renal disease (25). Our group has previously shown that syndecan-1 expression on tubular epithelium correlates with activation of renal repair mechanisms (26), and that syndecan-1 deficiency in human tubular epithelial cells leads to reduced proliferation (25). However, a direct role for syndecan-1 in complement activation has never been described.

Although the AP has been shown to play a role in numerous diseases, no specific inhibitor for the AP is yet available. Salp20 is a protein derived from the deer tick Ixodes scapulari and has been shown to inhibit the AP via the displacement of properdin from the alternative pathway C3 convertase. This causes an accelerated decay of the C3bBb complex and subsequent inhibition of the AP by up to 70% (27, 28). *In vivo*, treatment with Salp20 in mice showed a reduction of AP mediated damage in ovalbumine-induced asthma, elastase-induced abdominal aortic aneurysm and after intraperitoneal injections with LPS (29). However, to the best of our knowledge, no experiments have been performed which assess the inhibition of the pattern recognition of properdin using Salp20. Therefore, in this study we investigated the interactions of properdin with PTECs, followed by AP activation and the inhibitory effects of Compstatin, Salp20 and heparinoids. To investigate the binding capacity of properdin from different sources and subsequent AP activation, experiments were performed with either normal human serum as a source of properdin, biochemically purified properdin and recombinant full length properdin.

## Methods

### HK-2 Cells

The immortalized human kidney proximal tubular epithelial cell line HK-2 was obtained from ATCC (Manassas VA, USA). Cells were cultured in DMEM/F12 medium 1:1 (Invitrogen, Carlsbad, CA, USA), supplemented with 5 μg/ml insulin, 5 μg/ml transferrin, 5 μg/ml selenium, 36 ng/ml hydrocortisone, 10 ng/ml epidermal growth factor (All purchased from Sigma, Zwijndrecht, The Netherlands), and 50 U/ml penicillin, 50 μg/ml streptomycin and 25 mM Hepes (All purchased from Invitrogen, Carlsbad, CA, USA).

### Syndecan-1 Knockout Cell Line

Production of the syndecan-1 knockout HK-2 cell line by shRNA technology has been described before (25, 30, 31). To confirm syndecan-1 knockdown and to evaluate the binding of properdin to wild type HK-2 cells or syndecan-1 knockout HK-2 cells, syndecan-1 expression and properdin binding on wild-type and syndecan-1 deficient HK-2 was determined by flow cytometry. Cells were plated in 6-wells cell culture plates at 37°C and were detached using cell dissociation solution (C5789, Sigma®, Zwijndrecht, The Netherlands), 900 μL/well at 37°C until cells were detached. Cells were collected in 4.5 mL tubes containing 2 mL cell medium, centrifuged at 300 × g for 5 min at 4°C and washed with ice-cold phosphate-buffered saline (PBS)/1% bovine serum albumin (BSA) (FACS buffer) (Sigma®, Zwijndrecht, The Netherlands). Cells were subsequently incubated with Alexa Fluor®647 mouse anti-human anti-syndecan-1 (CD138; Bio-Rad/AbD Serotec, California, USA) antibody in FACS buffer on ice, or purified properdin (human factor P, Millipore, Cat 341283-250 1.1mg/mL) followed by rabbit anti-human properdin, prepared as described before (20), and goat anti-rabbit FITC (Southern Biotech, Birmingham, USA) in FACS buffer on ice in the dark. After washing, cells were resuspended in 300 μL of FACS buffer and analyzed in a FACSCalibur™ (FACSCalibur, Becton Dickinson, New Jersey, USA). The purified properdin used was stored at −80°C and thawed only once for the experiments. Non-relevant mouse IgG served as an isotype control. Experiments were independently repeated 4 times. The percentage reduction was calculated as reduction in % = 100–[(MFI KO × 100)/MFI wild type].

### Binding of Properdin to HK-2, Alternative Pathway Complement Activation and Inhibition by Compstatin

Properdin binding to the immortalized human kidney proximal epithelial cell line HK-2 was tested using properdin from various sources. Normal human serum (NHS) from a healthy volunteer with normal classical, alternative and lectin pathway activity, as detected by functional ELISA and determined before, was used as a serum properdin source (32). Concentrations of 5, 20, and 50% NHS diluted in DMEM/F12 culture medium were tested. Also purified properdin (human factor P, Millipore, Cat 341283-250 1.1 mg/mL) and recombinant full length properdin, produced in HEK E+ cells resulting in the same N-linked glycanation as plasma properdin, were used. Recombinant properdin had the physiological 1:2:1 ratio and did not contain non-physiological aggregates after purification. The recombinant properdin was aliquoted and stored at −80°C and thawed only once for the experiments (33).

Confluent HK-2 were cultured on a 6-well tissue culture plate and incubated for 36–48 h with 10 μg/ml Compstatin that was not exposed to freeze-thaw cycles before (a kind gift from professor J. D. Lambris, University of Pensylvania, Philadelphia, PA, United States) to prevent eventual C3b deposition. Cells were detached using cell dissociation solution (C5789, Sigma®, Zwijndrecht, The Netherlands), 900 μl/1mL at 37°C, collected in 4.5 mL tubes containing 2 mL cell medium and centrifuged twice at 250 g for 6 min at 20°C. For Compstatin mediated inhibition assays, cells were incubated with heat inactivated NHS in a serial dilution of 5, 20, or 50% NHS, purified properdin or recombinant properdin in the presence or absence of 10 μg/ml Compstatin for 30 min at 37°C. Hereafter, cells were centrifuged for 6 min at 250 g at 20°C. To detect bound properdin, cells were incubated with rabbit anti-human properdin (20), followed by goat anti-rabbit FITC (Southern Biotech, Birmingham, USA) in FACS buffer on ice in the dark.

To explore alternative pathway complement activation the same procedure as described above was followed. Subsequent to the incubation of properdin from NHS, purified properdin or recombinant properdin, cells were washed twice at 250 g without a break for 6 min at 20°C and were incubated in the presence or absence of 5% serum as a complement source for 45 min at 37°C. After incubation, cells were washed once with 2 mL 20°C FACS buffer at 250 g for 6 min at 4°C and once with 2 mL 4°C FACS buffer at 250 g without a break for 6 min at 4°C.

To detect activated C3, cells were incubated with mouse anti-human activated C3 recognizing C3b, iC3b, and C3c fragments (Clone bH6, HM2168S, Hycult biotech, Uden, The Netherlands) for 30 min on ice. Cells were washed twice with ice cold FACS buffer, centrifuged at 250 g for 6 min at 4°C, and incubated goat anti-mouse FITC (Purchased from Southern Biotech, Birmingham, USA) for 30 min on ice in the dark. Propidium iodide 1 μg/ml (Molecular Probes, Leiden, The Netherlands) was added just before measuring to be able to exclude apoptotic and necrotic cells. Properdin binding and activated C3 deposition on viable non-apoptotic cells were analyzed in a FACSCalibur™ (FACSCalibur, Becton Dickinson, New Jersey, USA). Results are from six independent experiments.

### Inhibition of Binding of Properdin to C3b

Competition ELISA was used to evaluate whether heparin-albumin (200 kDa), unfractionated heparin (15–18 kDa), low molecular weight (LMW)-heparin (4.5 kDa), C3b (185 kDa), and recombinant Salp20 (48 kDa) inhibit the binding of properdin to immobilized C3b. Heparin-albumin was from Sigma-Aldrich (Saint Louis, MO, USA). According to the data sheet, this artificial proteoglycan contained 4.8 moles heparin per mole albumin, with a protein content of 55%. Maxisorp 96-well flat bottom microtiter plates (U96 from Nunc International, Amsterdam, The Netherlands) were coated overnight with 1 μg/ml C3b in PBS at 4°C. C3b was purified as described before (34). After washing in PBS, wells were blocked with 1% BSA in PBS for 1 h at 37°C. A concentration range of heparin-albumin, unfractionated heparin, LMW-heparin, C3b or recombinant Salp20 was pre-incubated with 62.5 ng/ml properdin (Millipore, Billerica, Massachusetts, USA) in PBS, 0.05% Tween and 1% BSA for 15 min at room temperature. Thereafter the co-incubated heparin-albumin, unfractionated heparin, LMW-heparin, C3b and recombinant Salp20, together with properdin, was incubated on the C3b coated plate for 1 h at 37°C. Binding of properdin was detected with biotinylated rabbit anti-human properdin 1:3,000 diluted in PBS, 0.05% Tween and 1% BSA. After washing, streptavidin HRP (DAKO, Glostrup, Denmark) 1:5,000 was added to the plate and incubated for 1 h. Substrate reaction was performed with 3.3',5.5'-tetramethylbenzidine substrate (Sigma, Zwijndrecht, The Netherlands) for 15 min in the dark, and the reaction was stopped by adding 1.5 N H_2_SO_4_. Absorbance was measured at 450 nm in a microplate reader. All incubations were carried out in a volume of 100 μl/well. The experiment was independently repeated 3 times.

### Dose Dependent Block and Dose Dependent Competition of Properdin Binding to HK-2 by Recombinant Salp20

To evaluate whether recombinant Salp20 can inhibit the binding of properdin to the immortalized human kidney proximal epithelial cell line HK-2, cells were cultured in 6-well tissue culture plates. Cells were detached with cell dissociation solution as described previously, transferred into a 5 mL FACS tube with medium and centrifuged at 200 g for 7 min at 20°C. After washing, cells were incubated with a serial dilution of recombinant Salp20 of 0, 125, 250, 500, 1,000, or 8,000 ng/mL recombinant Salp20 together with 10 μg/mL purified properdin for 30 min at 37°C.

To evaluate whether recombinant Salp20 can dose-dependently compete off bound properdin and the activation of C3 and C5b-9 on HK-2, cells were also cultured in a 6-well tissue culture plates. Cells were detached with cell dissociation solution as described before, transferred into a 5 mL FACS tube with medium and centrifuged at 200 g for 7 min at 20°C. Cells were incubated with or without 10 μg/mL purified properdin (human factor P, Millipore, Cat 341283-250 1,1 mg/mL) for 30 min at 37°C. Cells were washed twice at 200 g for 7 min at 20°C and incubated with a serial dilution of pre-incubated recombinant Salp20 of 0, 32, 125, and 500 ng/mL together with 5% NHS for 1 h at 37°C.

To detect bound properdin, activated C3 or neoantigen C9 (as a measure of C5b-9 formation), cells were incubated with either rabbit anti-human properdin, mouse anti-human activated C3 (Clone bH6, HM2168S, Hycult biotech, Uden, The Netherlands), or with mouse anti-human neoantigen C9 (Clone WU13-15, HM2264, Hycult biotech, Uden, The Netherlands), for 30 min on ice. Cells were washed with ice cold FACS buffer, centrifuged at 250 g for 6 min at 4°C, and incubated with goat anti-rabbit FITC or goat anti-mouse FITC (both purchased from Southern Biotech, Birmingham, USA) for 30 min on ice in the dark. Propidium iodide 1 μg/ml (Molecular Probes, Leiden, The Netherlands) was added just prior to measurement in order to exclude apoptotic cells. Properdin binding and activated C3 deposition on non-apoptotic cells were analyzed in a FACSCalibur™ (FACSCalibur, Becton Dickinson, New Jersey, USA). Experiments were repeated independently two times. The percentage effect was calculated based on the control data (0 ng/mL Salp20 + 10 μg/mL purified properdin) in median fluorescence intensity (MFI), % inhibition = 100–(MFI test result/MFI control) × 100.

### Statistics

GraphPad Prism version 7.02 was used for statistical analyses. Data was examined by one-way ANOVA and the Mann-Whitney-U test and the Wilcoxon rank sum test were used as appropriate for the different experiments. A *P*-value <0.05 was considered statistically significant.

## Results

### Properdin From Various Sources Binds With PTECs *in vitro* and Functions as a Docking Station for Alternative Pathway Complement Activation

**Figure 1:**
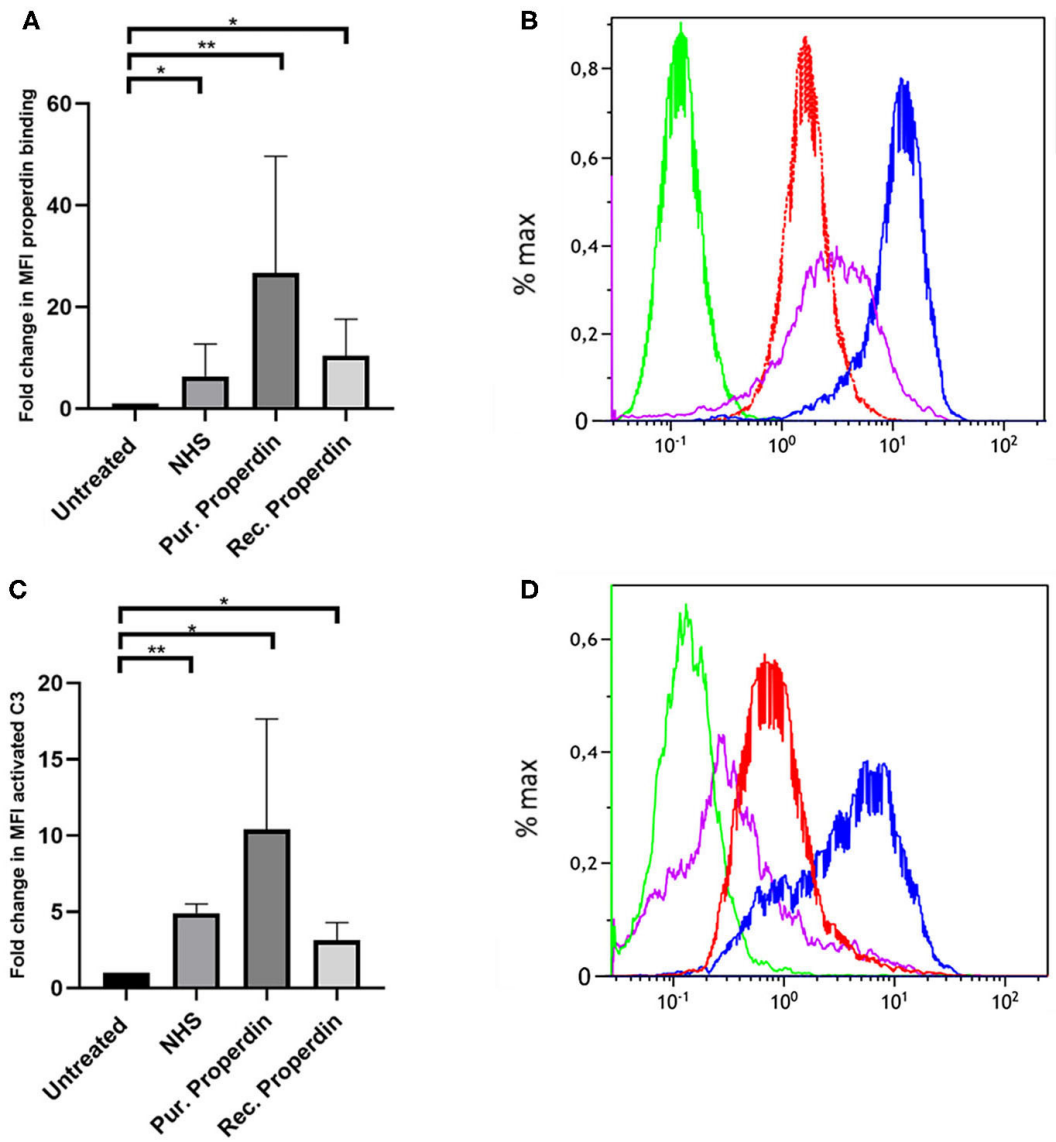
Properdin from various sources bind with PTEC *in vitro* and functions as docking station for alternative pathway complement activation. **(A)** Properdin present in 50% normal human serum (NHS), purified properdin and recombinant properdin show binding with PTEC in comparison to the negative control. Data presented as fold change compared to the untreated control and analyzed by the Wilcoxon trank sum test (*P < 0.05, **P < 0.01). Asterisks above the capped lines denote significant differences between the untreated samples and the properdin binding from different sources (*n* = 10). **(B)** Representative flow cytometry experiment for properdin binding from 50% NHS (red line), purified properdin (blue line) and recombinant properdin (purple line) in comparison the untreated sample (green line). Data represented as % max (data normalized for the peak at 100%). **(C)** Complement C3 activation via properdin from NHS, purified properdin and recombinant properdin shows complement activation via the alternative pathway. Data presented as fold change compared to the untreated control and analyzed as described in **(A)** (*n* = 6). **(D)** Representative flow cytometry experiment for complement C3 activation via NHS (red line), purified properdin (blue line), recombinant properdin (purple line) and the untreated sample (green line).

Properdin binding to PTECs using properdin present in normal human serum, purified properdin and recombinant properdin was tested to investigate differences in properdin binding and complement activation between different preparations. Incubation for 30 min with 50% normal human serum (NHS), purified properdin and recombinant properdin, resulted in substantial binding of properdin detected by flow cytometry. Untreated samples served as a control. Means in median fluorescence intensity (MFI) were 35, 158, 68, and 7, respectively. Binding of all preparations was statistically different (*P* = 0.02, *P* = 0.005, *P* = 0.049, respectively) when compared as fold change (fold change = MFI treated cells/MFI untreated cells) to the untreated control. No statistically significant differences were found between recombinant properdin vs. either purified properdin or NHS (*P* = 0.24 and *P* = 0.11). A significant difference was found between 50% NHS vs. purified properdin (*P* = 0.049) (Figures 1A,B). In the presence of NHS as a complement source, deposition of activated C3 followed the same pattern (Figures 1C,D). Means in MFI were 31, 72, 16, and 6 for NHS, purified properdin, recombinant properdin and the untreated sample, respectively. Activated C3 deposition were statistically different (*P* = 0.005, *P* = 0.01, *P* = 0.01, respectively) when compared as fold change (fold change = MFI treated cells/MFI untreated cells) to the untreated control for all preparations. No statistically significant differences were found for activated C3 deposition between purified properdin vs. recombinant properdin (*P* = 0.07), NHS vs. recombinant properdin (*P* = 0.06) or NHS vs. purified properdin (*P* = 0.17). As properdin may serve as the docking station for AP complement activation and to unravel the role of properdin in C3 activation on PTEC, we decided to further investigate complement activation on PTEC with and without C3 inhibitor Compstatin.

### AP Activation but Not Properdin Binding to PTECs Can Be Inhibited by Compstatin

**Figure 2:**
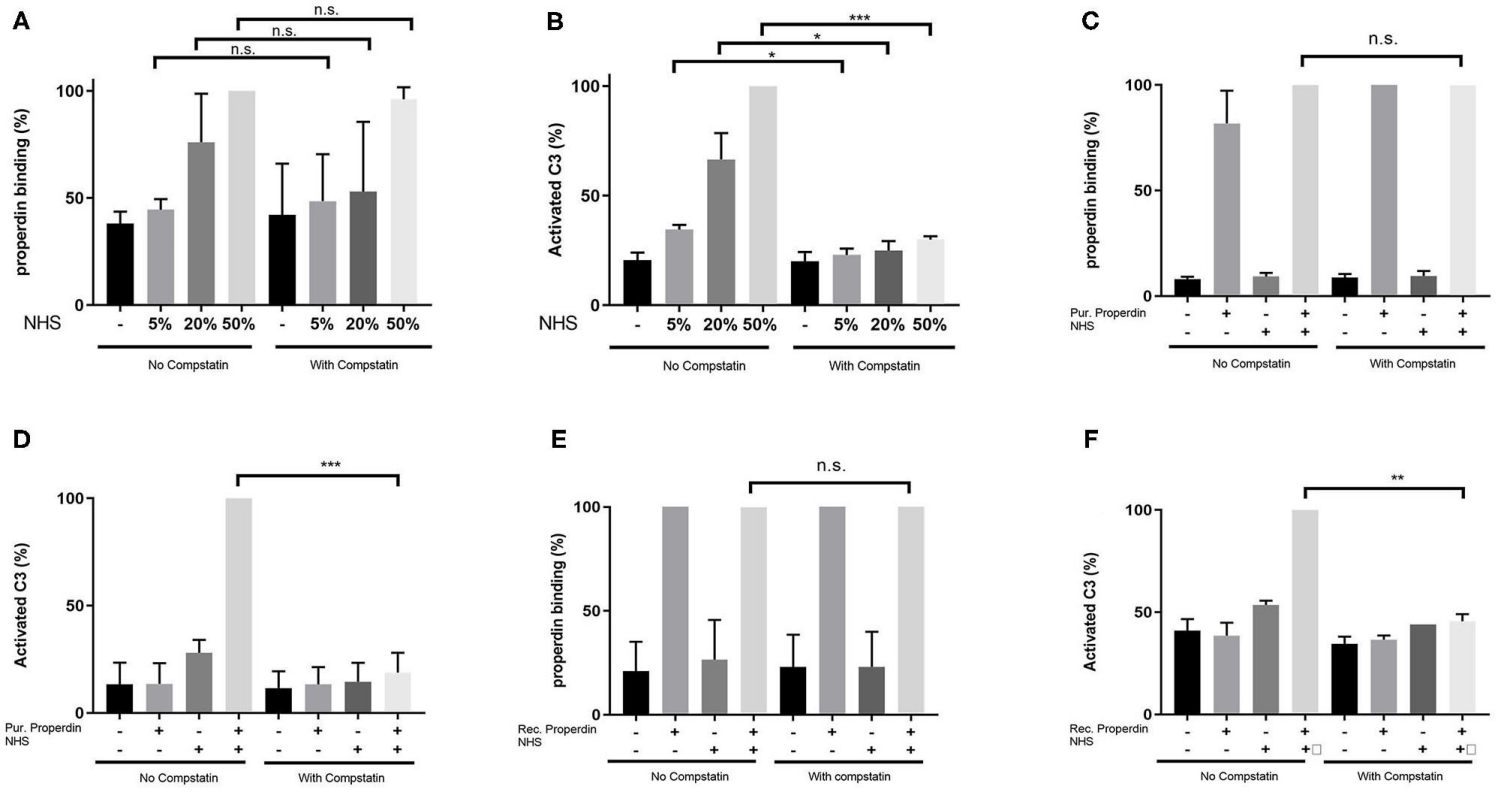
Properdin binding with PTECs *in vitro* is not mediated via C3b. **(A)** Properdin binding from serum is not influenced by co-incubation with compstatin. Bars represent a serial dilution of 5, 20, and 50% NHS. **(B)** C3 complement activation mediated by properdin from serum is completely inhibited by compstatin. **(C)** Properdin binding purified from plasma is not influenced by compstatin. **(D)** C3 complement activation by purified properdin is completely inhibited by compstatin. **(E)** Recombinant properdin binding is not influenced by compstatin. **(F)** C3 complement activation by recombinant properdin is inhibited by compstatin. All graphs are normalized by the sample without compstatin but with NHS **(A,B)** or purified properdin with NHS **(C,D)** or recombinant properdin with NHS **(E,F)**. Data were analyzed by the Mann-Whitney U test with an option of multiple comparison (*P < 0.05, **P < 0.01, ***P < 0.001). Asterisks above the capped lines denote significant differences between the sample without Compstatin but with NHS **(A,B)** or purified properdin with NHS **(C,D)** or recombinant properdin with NHS **(E,F)** and the same sample with Compstatin. *N* = 6 for all experiments.

HK-2 cells were incubated with Normal Human Serum (NHS) as a source of properdin in increasing concentrations of 5, 20, and 50%. Binding of properdin from serum to HK-2 was not affected by pre-incubation and co-incubation with the C3 inhibitor Compstatin, demonstrating that properdin binding with HK-2 cells is independent of the presence of C3b (5% NHS; *P* = 0.82, 20% NHS; *P* = 0.49 and 50% NHS; *P* = 0.42) (Figure 2A). Measurement of activated C3 deposition on HK-2 cells after incubation with NHS confirmed the functionality of Compstatin, since no increase in activated C3 deposition was seen during co-incubation with Compstatin (5% NHS; *P* = 0.04, 20% NHS; *P* = 0.04 and 50% NHS; *P* = 0.0002) (Figure 2B). This implies that properdin might act as a pattern recognition molecule on PTECs.

Similarly, when purified properdin or recombinant properdin was used as a source of properdin, pre-incubation and co-incubation of properdin with Compstatin did not affect the binding of properdin on HK-2 cells, further strengthening the finding that properdin binding to PTECs is independent of prior C3b deposition (*P* = 0.23 and *P* = 0.50, respectively) (Figures 2C,E). Co-incubation of serum with Compstatin after incubation of HK-2 cells with purified properdin or recombinant properdin resulted in the inhibition of activated C3 deposition, verifying the C3 inhibitory potential of Compstatin (*P* < 0.0001 and *P* = 0.002 respectively) (Figures 2D,F). In order to confirm the absence of C3 components (including C3b) on the PTEC cell surface to which properdin in NHS can bind, PTEC were stained for activated C3 (recognizing the cleavage fragments of C3b, iC3b, and C3c) before and after NHS treatment with and without pre-incubation with Compstatin (Supplementary Figure 1). C3 components were not detectable on untreated PTEC when compared to the background staining (*P* = 0.48). In conclusion, flow cytometry showed that properdin binds to HK-2 cells independent of C3b and regardless of properdin source.

### Binding of Properdin to Proximal Tubular Epithelial Cells Is Partly Mediated by Syndecan-1

**Figure 3:**
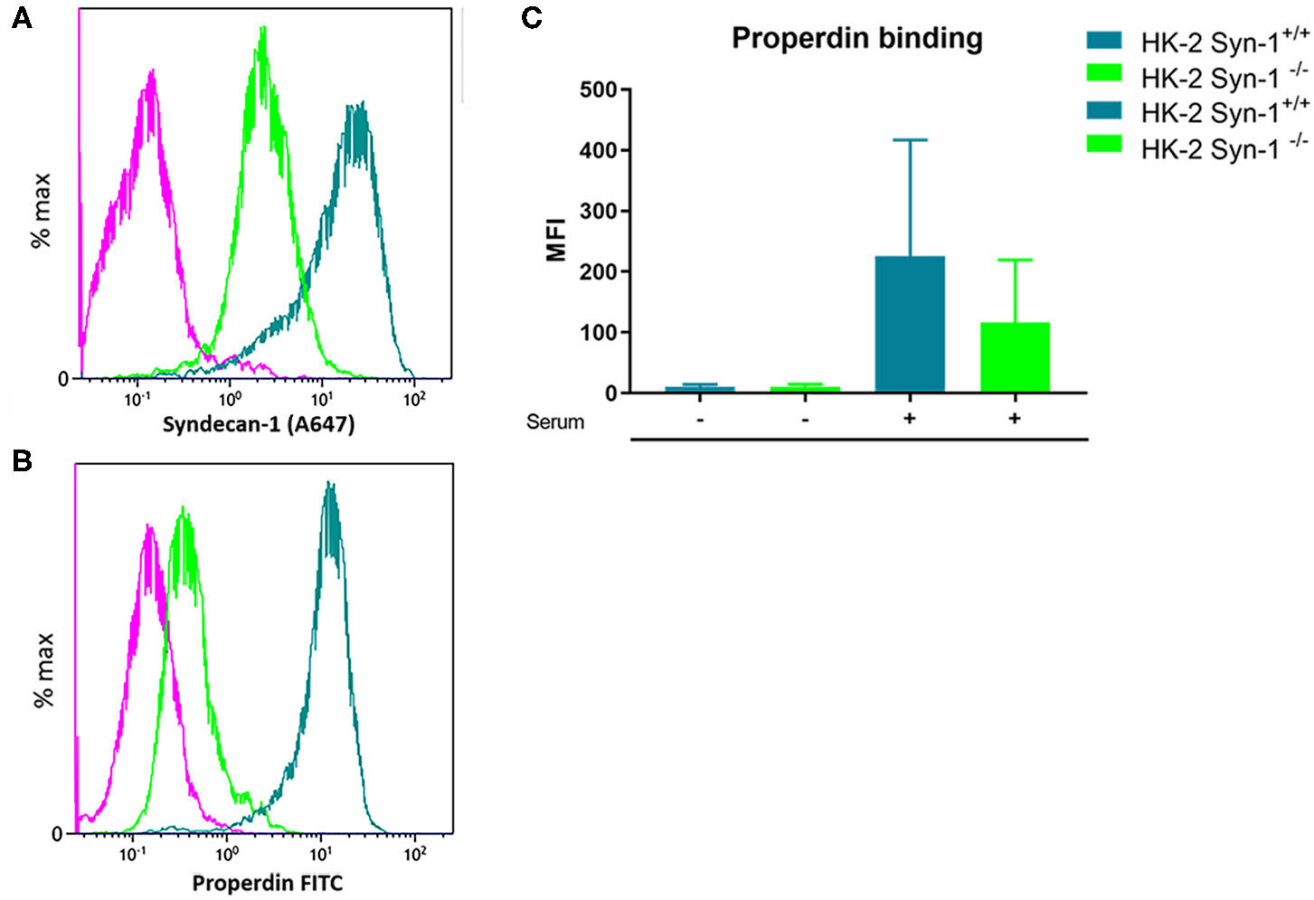
Properdin binding with PTEC *in vitro* is largely via syndecan-1 associated heparan sulfate. **(A)** A representative experiment showing high expression of syndecan-1 in PTEC wild type (turquoise) and >80% less expression of syndecan-1 in syndecan-1 KO PTEC (Green). **(B)** A representative experiment showing strong properdin binding to PTEC wilde type (turquoise) and ~75% less properdin binding to syndecan-1 KO PTEC (Green). The purple color represents the antibody binding of the isotype control. X-axis is a logarithmic scale, MFI noted in the figure. Data represented as % max (data normalized for the peak at 100%). **(C)** Syndecan-1 deficient cells show a reduction in properdin binding compared to HK-2 WT cells the difference was borderline significant (*n* = 4, *P* = 0.057). Data is expressed as mean ± SEM.

In former studies it was shown that heparitinase I treatment of HK-2 cells obliterated properdin binding, while immunofluorescent staining showed co-localization of properdin with syndecan-1 *in vivo* on tubular epithelium under nephrotic conditions (23). To identify the binding site of properdin on tubular cells, we tested properdin binding capacities of syndecan-1 silenced cells by short hairpin RNA technology. Stably transfected HK-2 *Synd1*^−/−^ cells showed ~80% reduction in syndecan-1 expression (Figure 3A). The HK-2 *Synd-1*^−/−^ cells show a ~70% reduced properdin binding potential compared to HK-2 WT cells(*P* = 0.057) (Figures 3B,C).

### AP Activation and Properdin Binding to PTECs Can Be Inhibited by Recombinant Salp20

**Figure 4:**
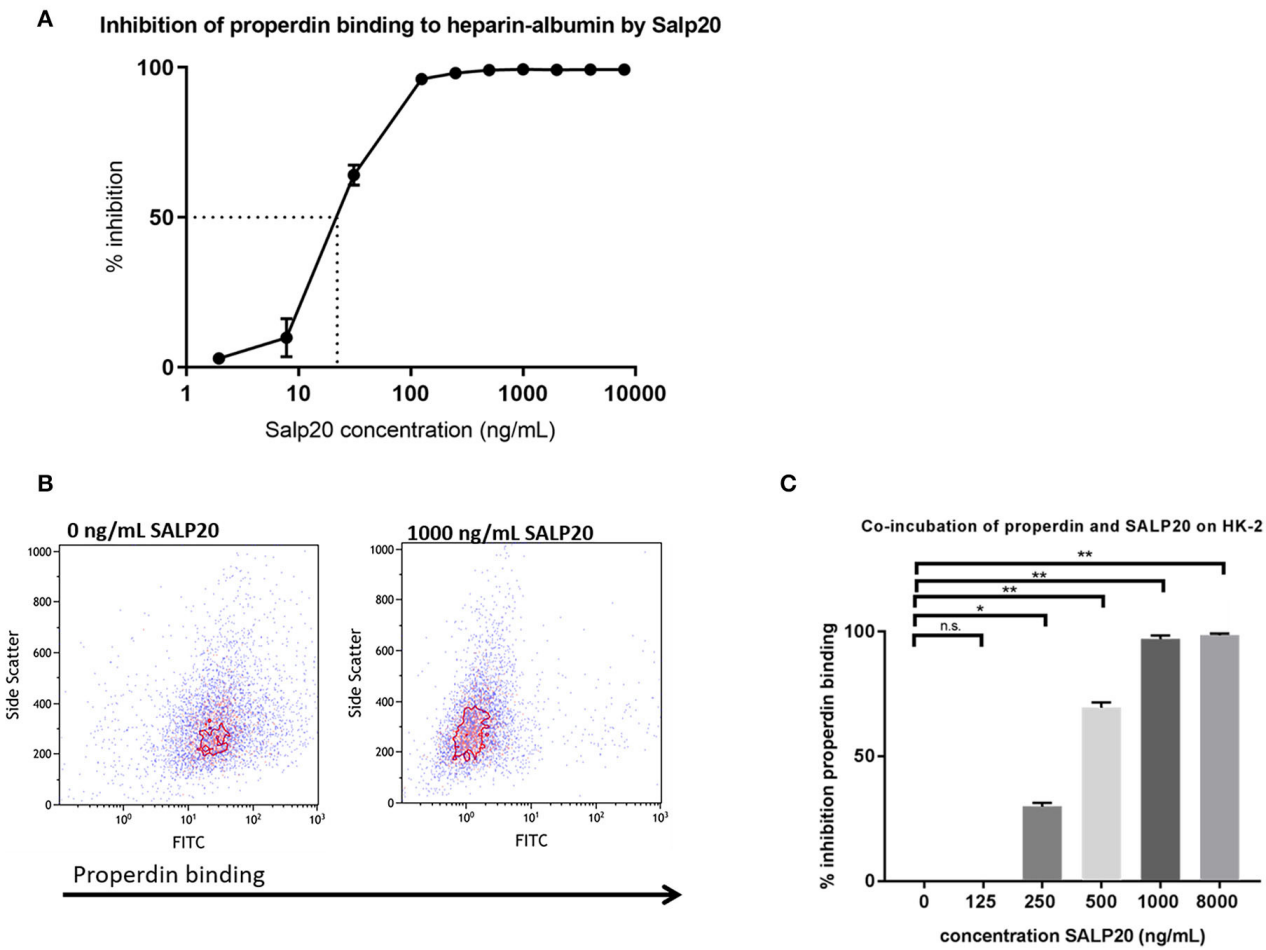
Properdin binding to heparin-albumin in ELISA and to HK-2 cells can be dose-dependently blocked by recombinant Salp20. **(A)** Pre-incubation of properdin with Salp20 dose-dependently reduced properdin binding to immobilized heparin-albumin. Dotted line in figure *A* represents the IC50. Experiments were independently repeated in duplicate. **(B)** A representative flow cytometry experiment shows that properdin co-incubation with 1,000 ng Salp20 reduces properdin binding to HK-2 in comparison to properdin incubation with HK-2 without SALP20. **(C)** Quantitative analysis of multiple experiments (*n* = 3) shows that recombinant Salp20 significantly blocks the binding of properdin to HK-2 per dose. Data were analyzed by Mann-Whitney U test with an option of multiple comparison (*P < 0.05, **P < 0.01). Asterisks above the capped lines denote significant differences between the untreated samples and the recombinant Salp20 inhibition with different concentrations.

It has been described that Salp20 (a deer tick protein) functions as a properdin-blocking agent, displacing properdin from the C3-convertase (27). To evaluate whether Salp20 inhibits the binding of properdin to HSPGs, resulting in inhibition of properdin's pattern recognition capacity of HSPGs, we co-incubated recombinant Salp20 with properdin in absence of activated C3 and measured the binding of properdin to heparin-albumin. Increasing concentrations of recombinant Salp20 showed dose dependent inhibition of properdin to heparin-albumin with an IC50 of 18 ng/ml (Figure 4A). Thus, showing that Salp20 indeed inhibited the binding of properdin to an HSPG analog, abolishing pattern recognition of HSPGs by properdin.

Recombinant Salp20 was also tested for properdin binding and AP inhibitory potential on HK-2 cells by flow cytometry. Incubation of 10 μg/mL purified properdin, in the absence of activated C3 conditions, led to properdin deposition on the HK-2 cells, while recombinant Salp20 led to a dose dependent reduction in binding of purified properdin to the cells (Figures 4B,C). An inhibitory effect of 70% was achieved when incubating the cells with 500 ng/ml recombinant Salp20 (*P* = 0.01) and an inhibitory effect of 98% (*P* = 0.0003) when incubating the cells with >1,000 ng/ml recombinant Salp20 (Figure 4C).

**Figure 5:**
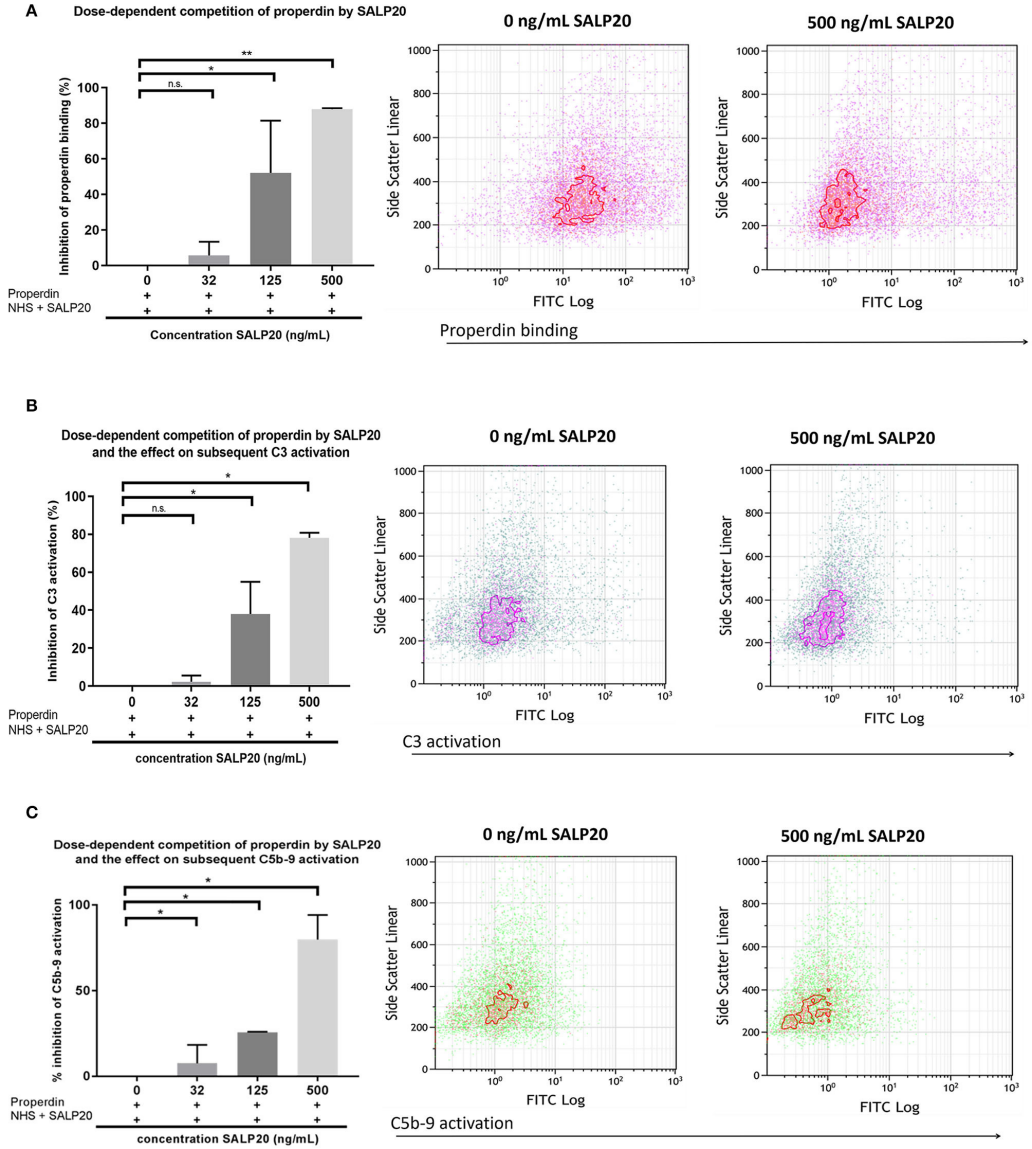
Recombinant Salp20 dose-dependently competes with HK-2 bound properdin, thereby preventing AP complement activation. **(A)** PTECs were incubated with purified properdin followed by incubation with NHS and increasing concentrations of Salp20. Bars show quantitative analysis of multiple experiments (*n* = 2). Density plots of a representative experiment show the shift in PTEC-bound properdin in the presence of 500 ng/ml Salp20. **(B)** C3 activation can be dose dependently inhibited by recSALP20. The same experimental set up as in **(A)**, but now C3 activation was detected. **(C)** C5b-9 activation can be dose dependently inhibited by Salp20. The same experimental set up as in **(A,B)**, but now C5b-9 activation was detected. Data were analyzed by Mann-Whitney U test with an option of multiple comparison (*P < 0.05, **P < 0.01). Asterisks above the capped lines denote significant differences between the untreated samples and the recombinant Salp20 inhibition with different concentrations.

The dose dependent capacity of recombinant Salp20 to displace cell-bound properdin was also tested by flow cytometry. After pre-incubation with purified properdin, HK-2 cells were incubated with an increasing concentration of up to 500 ng/ml of Salp20 and NHS. Recombinant Salp20 dose-dependently displaced bound properdin with an inhibitory effect of 90% with 500 ng/ml Salp20 (*P* = 0.003) (Figure 5A). Recombinant Salp20 was also effective in the inhibition of AP activation, shown by dose-dependent reduction of activated C3 and C5b-9 deposition. Concentration dependent reduction of activated C3 and C5b-9 deposition by recombinant Salp20, showed a similar pattern compared to properdin binding (Figures 5B,C). The maximum concentration of recombinant Salp20, 500 ng/ml, resulted in an inhibition of 80% in activated C3 deposition (*P* = 0.02) and 90% in C5b-9 deposition (*P* = 0.006) compared to the non-inhibited control.

### Inhibition of Properdin to C3b by Heparins, C3b and Recombinant Salp20

In the experiments described up to now we evaluated the properdin – syndecan-1/HS interaction as a focus point for intervention. In a last series of experiments, we evaluated the properdin-C3b interaction as a target point. Thus, we set out a series of competition experiments using the potential dose-dependent inhibitory activity of heparins, C3b or recombinant Salp20 on properdin binding to immobilized C3b. The results of the binding assay showed that heparin-albumin, unfractionated heparin, C3b and recombinant Salp20 inhibit properdin binding to C3b in a dose-dependent manner. (IC50 8.6 ng/mL for heparin-albumin, 63.4 ng/mL for unfractionated heparin, >1,000 ng/mL for LMW-heparin, 6,484 ng/mL for C3b and 33.5 ng/mL for recombinant Salp20). Also, the inhibitory capacity depends on the size of the heparin products (IC50 0.000043 nmol/mL for heparin-albumin, 0.004 nmol/mL for unfractionated heparin, >22.2 nmol/mL for LMW-heparin). Heparin has a lower IC50 compared to C3b (IC50 0.004 nmol/mL vs. IC50 0.035 nmol/mL). Recombinant Salp20 has a lower IC50 compared to heparin (IC50 0.0007 nmol/mL vs. IC50 0.004 nmol/mL, respectively) (Table 1). These data show that unfractionated heparin and recombinant Salp20 not only compete for properdin binding with heparan sulfates, but also for properdin binding with C3b.

**Table 1:** Inhibition of properdin binding to C3b; *IC*_50_, Concentration causing 50% properdin binding inhibition expressed in ng/mL and nmol/mL.

**Coating**	**Inhibitor**	**IC50 ng/mL**	**IC50 nmol/mL**
C3b	Heparin-albumin	8.6 ng/mL	0.000043 nmol/mL
	Unfractionated heparin	63.4 ng/mL	0.004 nmol/mL
	LMW-heparin	>1,000 ng/mL	>22.2 nmol/mL
	C3b	6,484 ng/mL	0.035 nmol/mL
	Salp20	33.5 ng/mL	0.0007 nmol/mL

*IC_50_ was calculated by non-linear regression with curve fitting in GraphPad Prism*.

## Discussion

In this study we provide evidence that properdin functions as a pattern recognition protein on PTECs where the binding is largely mediated via syndecan-1 associated heparan sulfate and is C3b-independent. Furthermore, we show that the tick protein Salp20 effectively blocks the heparan sulfate mediated pattern recognition by properdin, pointing toward the potential for therapeutic interventions at the tubular level in proteinuric conditions.

It has long been assumed that properdin, next to its stabilizing role of the alternative C3 convertase, could act as a pattern recognition molecule. We have shown previously that during proteinuria, properdin recognizes and binds to heparan sulfate proteoglycans (HSPG) on tubular epithelial cells (23). Our results in this study using the C3 inhibitor Compstatin, show that Compstatin can inhibit complement activation and therefore C3b deposition, but cannot preclude the deposition of properdin on PTECs. We display that this finding does not materially differ between properdin sources, including recombinant properdin. The latter confirms that our finding is robust, since different properdin isolates might differ in purity, conformation, multimerization, post-translational modifications, and the eventual presence of other co-purified or properdin-bound proteins. However, properdin in purified preparations is prone to aggregation which can be avoided to a certain extend by storage at 4°C for up to 2 weeks without any additional freeze/thaw cycles (12, 35). Our properdin preparations were exposed to one freeze/thaw cycle and our preparations could therefore contain non-physiological aggregates. However, since unpurified properdin present in serum essentially showed the same binding to PTECs as purified and recombinant properdin, it is unlikely that properdin aggregates importantly contributed to our findings. Therefore, irrespective of the source, all experiments indicate a real C3b-independent binding of properdin to heparan sulfates (HS), most likely present on the PTEC cell membrane as the polysaccharide side chains of syndecan-1. These findings are in agreement with experimental studies in C3 knockout mice demonstrating C3 deposition in glomeruli of mice with anti- GBM disease, although the authors did not address the glomerular cell type to which properdin binds (19).

Conversely, Harboe et al. recently showed that properdin binding on endothelial cells and *Neisseria meningitidis* is dependent on initial C3 deposition (18). Consequently, our findings reopen the discussion whether properdin is a true pattern recognition molecule of the alternative pathway (AP). Pattern recognition of properdin has been indicated in other properdin interactions as well. Properdin binding to DNA and glycosaminoglycans on late apoptotic cells and necrotic cells has been suggested to be independent of initial C3 deposition (16, 17). Glycosaminoglycans and DNA share a strong negative charge, while properdin is strongly positively charged. Therefore, the interaction of properdin with glycosaminoglycans (and DNA), is based on charge - charge interactions, as we previously analyzed in detail (23).

Syndecan-1 and properdin co-localize on PTECs under proteinuric conditions (23). We present that syndecan-1 may be a ligand of properdin, using a syndecan-1 deficient HK-2 strain. Syndecan-1 is a major membrane spanning HSPG in epithelial cells and the interaction of properdin with sulfated glycosaminoglycans has been long known. In this study we found a reduced binding of properdin in syndecan-1 deficient HK-2 cells when compared to HK-2 wild type cells. It is likely that properdin not only binds to syndecan-1 but also to other epithelial HSPGs of which syndecan-1 is the most important properdin binding HSPG. Properdin consists of seven non-identical trombospondin-1 repeats (TSR), and literature has shown that a fragment consisting of TSR 4 & 5 forms the binding site for glycosaminoglycans, but also for C3b (30). Earlier work already showed that trypsin treatment of properdin, cleaving the TSR5 in half, results in an inability to bind C3b while the glycosaminoglycan binding remains intact. This suggests that TSR5 is the principal C3b binding site for properdin that receives a co-operative contribution from TSR4 (30, 36). Recent structural studies revealed TSR5 to be the dominant C3b binding domain with some contribution of TSR6 (33). Taken together, these studies showed that the binding site for C3b and glycosaminoglycans on properdin could be different, but are more likely very close. We show that inhibition of properdin to C3b by different heparinoid products is size dependent; the bigger the better, suggesting that larger heparins sterically hinder the C3b binding site on properdin as well. We also show that the deer tick protein Salp20 can inhibit both the binding of heparin-albumin and C3b to properdin. Salp20 has previously been shown to displace properdin from the alternative C3 convertase, resulting in accelerated decay of the convertase (27). Our results confirm that recombinant Salp20 can inhibit the binding of properdin to C3b and thereby reduce the AP activation on PTECs. However, we also show that recombinant Salp20 can inhibit the binding of properdin to heparin-albumin and to HS on PTECs, indicating a double inhibitory role for Salp20 in properdin mediated AP activation, namely inhibition of the active C3 convertase and inhibition of the initial pattern recognition function of properdin. The results further strengthen the data shown by others that C3b and glycosaminoglycans have a closely related binding epitope on properdin (27). Nevertheless, further molecular docking studies are needed to unravel the exact glycosaminoglycan-binding domain of properdin.

It has been demonstrated before that Salp20 can inhibit the AP of complement in multiple disease models (29, 37). This could be of major importance in the development of therapeutic modalities for tubular damage in proteinuric renal diseases. Our results demonstrate that in this setting, recombinant Salp20 may not only block the binding of properdin to proximal tubular epithelial cells, but also compete off properdin that was already bound to the cells and avoid AP activation, even when the initial pattern recognition step had already formed. Since properdin-deficient humans do not show a severely compromised immune function, apart from an increased risk for meningitis for which vaccination is possible, blocking properdin seems a relatively safe approach (38, 39). There are some *in vitro* and *ex vivo* studies describing properdin competing composites and properdin blocking antibodies in humans, however unexpected results in animal models teach us that not all lessons have yet been learned (30, 40–45). Bansal-Gupta et al. described a properdin targeting monoclonal antibody, showing exclusive AP blocking activity by influencing the interaction of C3 with properdin in an *in vitro* model (40). In addition, Pauly et al. reported the development of an anti-properdin monoclonal antibody that showed up to fifteen times more efficiency in blocking the complement cascade when compared to anti-Ba or anti-C5 antibodies in human blood samples (41). Currently one anti-properdin antibody is at the stage of a phase 2 clinical trial (42). However, properdin doesn't spill the beans that easily. In contrast to the studies described above, a protective role for properdin was described in two separate C3 glomerulopathy (C3G) mouse models. This is remarkable, as C3G occurs as a result of overactivity of the AP, leading to glomerular injury. Nonetheless, mice knocked out for properdin (and small amounts of truncated factor H) showed an injury exacerbation with increased accumulation of C3 along the glomerular basal membrane (44, 45). In conclusion, discovering the role of properdin remains challenging and not fully elucidated.

**Figure 6:**
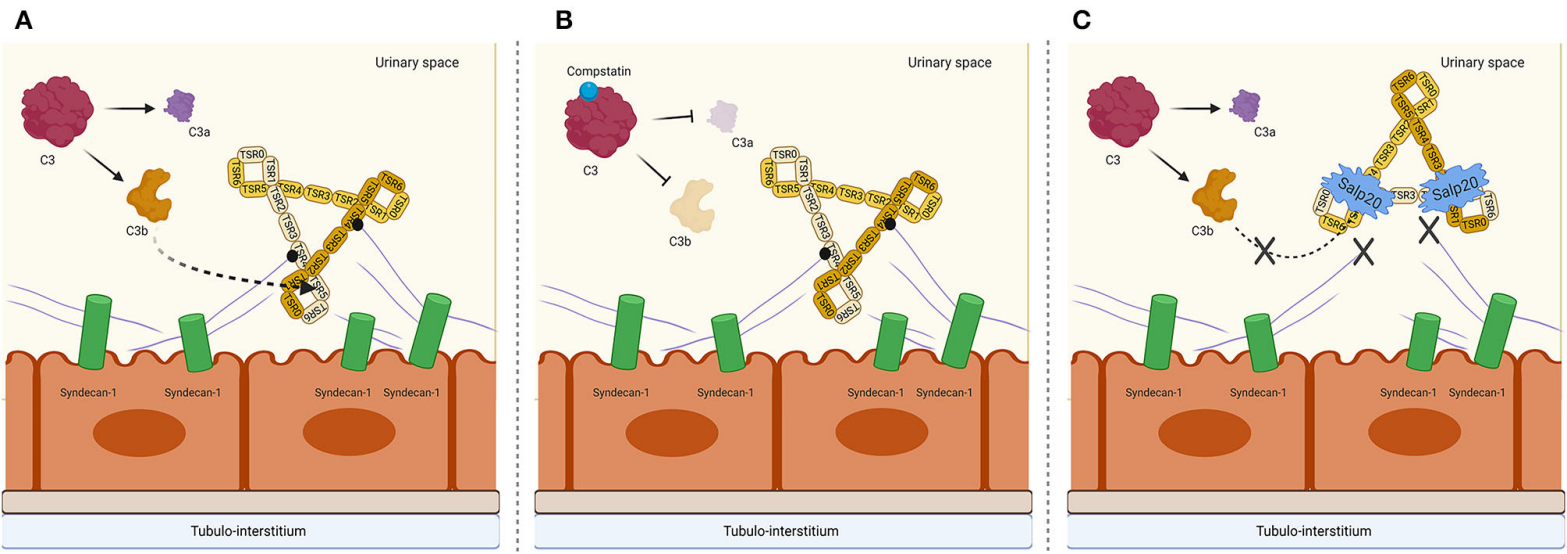
Proposed mechanism on a tubular epithelial level of properdin binding to syndecan-1/heparan sulfate and interruption of AP complement activation by C3 cleavage inhibiting peptide Compstatin and properdin inhibitor Salp20. **(A)** Properdin is depicted in its trimeric form since this is the most abundant form of properdin. The binding of syndecan-1 is most likely to the trombospondin-1 repeat (TSR) 4 domain of properdin, receiving a cooperative contribution of TSR5. C3b most likely binds to TSR5, with a cooperative function of TSR4 and TSR6. **(B)** In the presence of Compstatin, C3 cleavage is inhibited. However, properdin is able to bind to heparan sulfate proteoglycan syndecan-1. **(C)** In the presence of Salp20, C3b cannot bind to the TSR5 domain of properdin, nor can properdin bind to syndecan-1. Figure created with BioRender.com.

Salp20 is a new kid on the block in the field of properdin inhibition and since Salp20 is a tick protein, it would be expected to be strongly immunogenic. Therefore, prior to testing in animal models, small non-immunogenic molecule analogs of the Salp20 binding region should be produced and tested *in vitro* and *in vivo* for their AP inhibiting potential. Next to the inhibitory effect of Salp20, we have also shown in previous work that heparinoids compete for properdin binding with heparan sulfates on PTECs (24), and in that study we showed that non-anti-coagulant heparins also compete for properdin binding with C3b. This is promising for AP-driven diseases such as tubular activation secondary to proteinuria. Finally, we also show that Compstatin does not inhibit the binding of properdin to PTEC, but does prevent subsequent AP complement activation. Therefore, Compstatin holds promise in blocking undesired complement activation in numerous pathogenic conditions. A possible mechanism of AP complement inhibition on a tubular level by Compstatin and Salp20 is depicted in Figure 6. Overall, our study demonstrates the inhibitory effects of Compstatin, non-coagulant heparins and recombinant Salp20 at the level of proximal tubular epithelial cells. These results might be of great importance for reducing proteinuria induced AP activation and tubular injury.

## Data Availability Statement

The raw data supporting the conclusions of this article will be made available by the authors, without undue reservation.

## Author Contributions

RL was involved in study design, carrying out assays, interpreting data, statistical analysis, creating tables and figures, and writing of the manuscript. DT was involved in study design, interpreting data, and writing of the manuscript. WD was involved in carrying out assays and manuscript editing. MD and MS were involved in interpreting data and manuscript editing. SB and JB were involved in study design, interpreting data, statistical analysis, and manuscript editing. All authors contributed to the article and approved the submitted version.

## Conflict of Interest

The authors declare that the research was conducted in the absence of any commercial or financial relationships that could be construed as a potential conflict of interest.
