# Supplementary material for: Properdin Pattern Recognition on Proximal Tubular Cells Is Heparan Sulfate/Syndecan-1 but Not C3b Dependent and Can Be Blocked by Tick Protein Salp20
Source: Front Immunol. 2020 Aug 7;11:1643. doi: 10.3389/fimmu.2020.01643 (PMC7426487; doi:10.3389/fimmu.2020.01643)
Supplement: Supplementary file 1 [file Data_Sheet_1.PDF]

Online supplemental Results

Properdin pattern recognition on proximal tubular cells is heparan sulfate/ syndecan-1 but not C3b dependent and can be blocked by tick protein Salp20

Rosa G.M. Lammerts[1], Ditmer T. Talsma[1], Wendy Dam[1], Mohamed R. Daha[1], Marc A.J. Seelen[1], Stefan P. Berger[1], Jacob van den Born[1], on behalf of the COMBAT Consortium

1 *Division of Nephrology, Department of Internal Medicine, University Medical Center Groningen, University of Groningen, Groningen, The Netherlands*

Correspondence and request for reprints to:

Rosa G.M. Lammerts, M.D.

Division of Nephrology, Department of Internal Medicine

University Medical Center Groningen

Hanzeplein 1, Z2.18, 9713 GZ Groningen

Email: r.g.m.lammerts@umcg.nl

Supplementary Figure 1. C3 components C3b, iC3b and C3c (activated C3) are not detectable on the cell membrane of untreated PTEC.

PTEC were treated with NHS with and without pre-incubation with Compstatin. FACS staining for activated C3 on untreated PTEC (- - + +) does not show presence of C3 compared to the background staining without detecting antibody (- - - -) (P=0.48). Data presented as mean fluorescence intensity (MFI). Asteriks above the capped lines denote significant differences.
