# Supplementary figures and images for: Properdin Pattern Recognition on Proximal Tubular Cells Is Heparan Sulfate/Syndecan-1 but Not C3b Dependent and Can Be Blocked by Tick Protein Salp20
Source: Front Immunol. 2020 Aug 7;11:1643. doi: 10.3389/fimmu.2020.01643 (PMC7426487; doi:10.3389/fimmu.2020.01643)

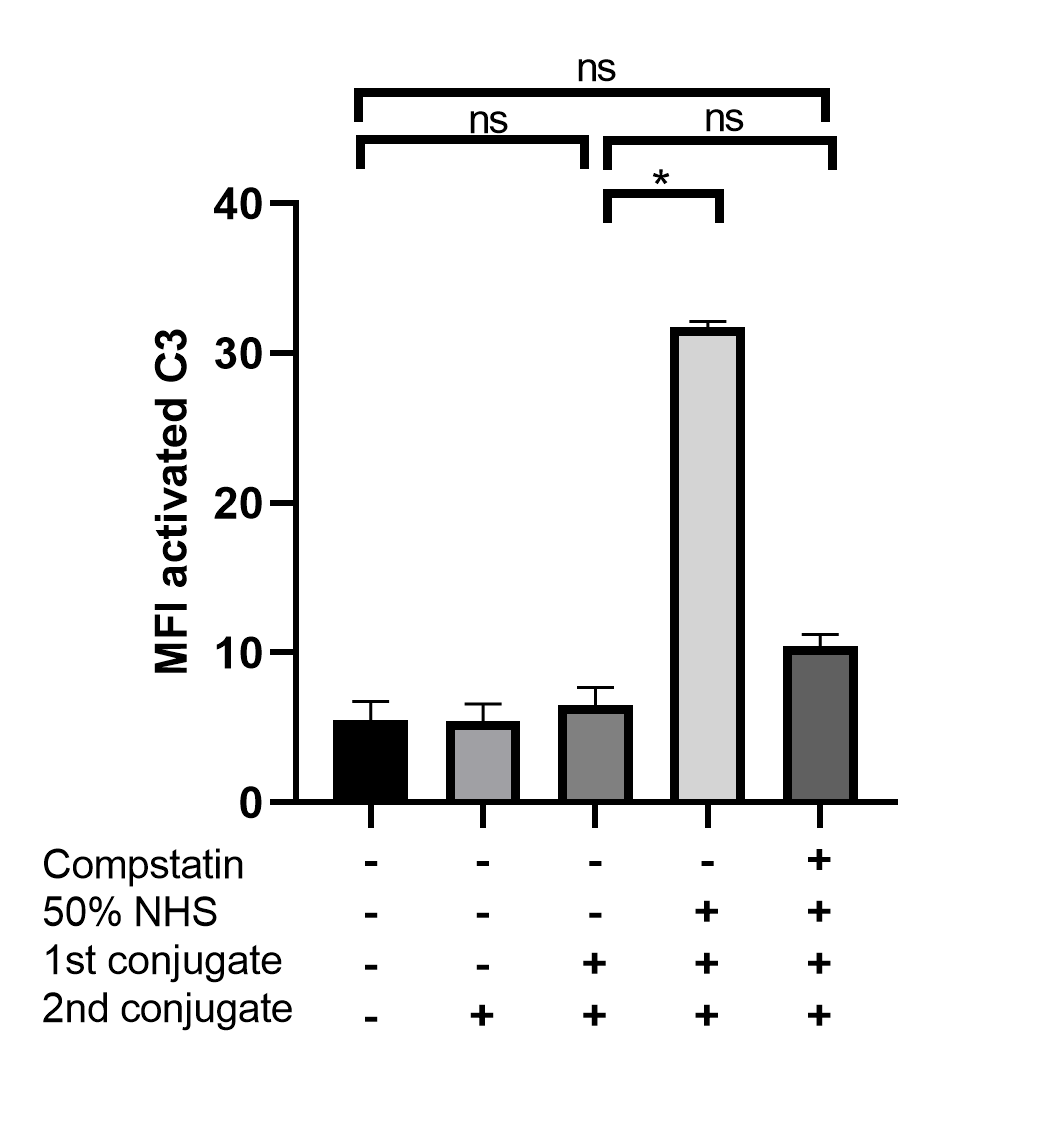

Supplement: Supplementary file 2 [file Image_1.tif]
